# Upstream Freshwater and Terrestrial Sources Are Differentially Reflected in the Bacterial Community Structure along a Small Arctic River and Its Estuary

**DOI:** 10.3389/fmicb.2016.01474

**Published:** 2016-09-21

**Authors:** Aviaja L. Hauptmann, Thor N. Markussen, Marek Stibal, Nikoline S. Olsen, Bo Elberling, Jacob Bælum, Thomas Sicheritz-Pontén, Carsten S. Jacobsen

**Affiliations:** ^1^Center for Biosustainability, Technical University of DenmarkHoersholm, Denmark; ^2^DTU Bioinformatics, Technical University of DenmarkKgs. Lyngby, Denmark; ^3^Center for Permafrost, University of CopenhagenCopenhagen, Denmark; ^4^Department of Ecology, Faculty of Science, Charles UniversityPrague, Czech Republic; ^5^Chr. Hansen A/SHoersholm, Denmark; ^6^Department of Environmental Science, Aarhus UniversityRoskilde, Denmark

**Keywords:** biodiversity, bacterial community, freshwater network, Greenland, arctic, polar environments

## Abstract

Glacier melting and altered precipitation patterns influence Arctic freshwater and coastal ecosystems. Arctic rivers are central to Arctic water ecosystems by linking glacier meltwaters and precipitation with the ocean through transport of particulate matter and microorganisms. However, the impact of different water sources on the microbial communities in Arctic rivers and estuaries remains unknown. In this study we used 16S rRNA gene amplicon sequencing to assess a small river and its estuary on the Disko Island, West Greenland (69°N). Samples were taken in August when there is maximum precipitation and temperatures are high in the Disko Bay area. We describe the bacterial community through a river into the estuary, including communities originating in a glacier and a proglacial lake. Our results show that water from the glacier and lake transports distinct communities into the river in terms of diversity and community composition. Bacteria of terrestrial origin were among the dominating OTUs in the main river, while the glacier and lake supplied the river with water containing fewer terrestrial organisms. Also, more psychrophilic taxa were found in the community supplied by the lake. At the river mouth, the presence of dominant bacterial taxa from the lake and glacier was unnoticeable, but these taxa increased their abundances again further into the estuary. On average 23% of the estuary community consisted of indicator OTUs from different sites along the river. Environmental variables showed only weak correlations with community composition, suggesting that hydrology largely influences the observed patterns.

## Introduction

Arctic river and estuary ecosystems are vulnerable to the ongoing climate change. Increasing temperatures are resulting in negative mass balance of glaciers and increased precipitation, with significant impacts on rivers and estuarine systems (Serreze et al., 2000; Mueller et al., 2003). In addition, Arctic rivers are known to transport significant amounts of organic carbon and biomass from permafrost and glacier ecosystems into the Arctic oceans and are therefore important factors in global climate change models (Kling et al., 1991; Guo et al., 2007; Lawson et al., 2014; Hawkings et al., 2015). Higher river flow associated with the warming climate may result in a more river-dominated community in the estuaries (Fortunato et al., 2013). Once riverine bacteria reach the estuary, they may influence local nutrient cycling through biofilm formation and forming aggregates (flocs; Decho, 2000). Thus, bacterial communities dispersed through Arctic riverine systems may be important for biogeochemical cycling processes in Arctic estuarine and coastal ecosystems.

There are a number of studies on Arctic estuarine ecosystems focused on biodiversity, biological productivity, seasonal variability, food web interactions, and responses to environmental variables (Galand et al., 2006, 2008; Wells et al., 2006; Vallieres et al., 2008; Fortunato et al., 2012, 2013). However, the river communities have usually been assessed as a whole. How different communities added to the river affect the estuarine community has not been addressed to date. While previous studies have shown that increased river flow alters Arctic river and estuary communities in seasonal patterns (Crump et al., 2009; Fortunato et al., 2012, 2013), it is yet unknown how upstream sources of freshwater microbial communities influence these communities.

A few recent studies on freshwater ecosystems at different spatial scales have greatly increased our understanding of the biogeography of riverine networks (Nelson et al., 2009; Crump et al., 2012, 2007; Ruiz-González et al., 2015; Niño-García et al., 2016). These studies have shown that biogeographic patterns of bacterioplankton communities are a result of the interaction between local environmental variables and mass-effects. Furthermore, that mass-effects are determined by the hydrology as well as the position along the network (Crump et al., 2007, 2012; Nelson et al., 2009; Ruiz-González et al., 2015; Niño-García et al., 2016).

Water residence time (WRT) has shown to be an important factor for determining the relative influence of hydrology vs. local sorting (Niño-García et al., 2016). There seems to be a greater influence from hydrology and mass-effects in systems with short WRT and a greater influence from local sorting in systems with long WRT (Niño-García et al., 2016). Longer WRT in lakes and larger rivers compared to smaller streams consequently results in less diverse communities due to local sorting (Niño-García et al., 2016). In this way, hydrology and local sorting interact and result in a uni-directional pattern of gradually decreasing diversity from smaller streams to larger rivers and lakes (Niño-García et al., 2016). Furthermore, beyond a WRT of 10 days hydrology has been shown to have no additional impact on the structuring of the microbial community (Niño-García et al., 2016). The importance of WRT for selecting lake-specific phylotypes in a freshwater network was also highlighted in another study (Nelson et al., 2009). The study showed less similarity between the microbial community in the inlet and the outlet of a headwater lake compared to the inlets and the outlets of downstream lakes. This indicates that the first lake selects for a lake-specific community, which is then transported downstream in the network (Nelson et al., 2009). These results also illustrated the importance of the position along the network for understanding the bacterial community structure (Nelson et al., 2009). As the position of water bodies in the system might be a key factor for determining the structure of the microbial community at that particular position, the right spatial resolution is important for understanding the structural changes the microbial community undergoes along a freshwater network.

Together with WRT a terrestrial seed bank for freshwater networks also seem to result in a uni-directional structure of the microbial community (Crump et al., 2012; Ruiz-González et al., 2015). In the catchment of the Toolik Lake, Alaska, a clear pattern of decreasing diversity was shown from soil waters farthest upstream with highest species richness through headwater streams and lastly to lowest richness in lake water (Crump et al., 2012). OTUs originating in soil were numerically dominant throughout a freshwater network in the Eastern boreal region of Québec, Canada, and certain OTUs that were rare in soil were shown to increase in number and become dominant in the downstream freshwater environments (Ruiz-González et al., 2015). These studies indicate that an initial inoculation from soil at the beginning of a freshwater network is followed by a species-sorting process downstream (Crump et al., 2012; Ruiz-González et al., 2015).

On a large spatial scale the uni-directional pattern of decreasing microbial diversity along a river might be explained by the common origin from a highly diverse terrestrial community (Ruiz-González et al., 2015) and by increasing local sorting (Niño-García et al., 2016). However, there might be another pattern on a smaller spatial scale revealed with higher resolution. Higher resolution of samples along a freshwater network might reveal the input of new microbial taxa of different origin along the freshwater network. Input of new taxa along a network could result in a different structuring pattern of the microbial community, which is not uni-directional. The addition of new microbial communities along a freshwater network would be particularly clear in smaller networks where input makes up a larger fraction of the downstream water body. This also implies that in larger networks, the downstream community might mask new communities added along the network. Therefore, river communities on a small spatial scale may show not to have a uni-directional structure, explained by seeding with new microbial communities along the network. Furthermore, it is yet unknown whether the spatial directionality described above extends into saline waters or whether the very different environmental conditions met by the riverine community in the estuary result in a different pattern of the microbial network.

We address the question of how the bacterial communities from the Red River, a small river on the Disko Island, West Greenland (69°N) are structured at the small spatial scale, by comparing bacterial communities from five sites along the river including input sites from a glacier and a proglacial lake. We investigate whether the structure of the riverine bacterial communities can help explain the structure of the estuary communities by including 23 samples through three transects of the Red River estuary.

Sampling was done in 2013 in August when precipitation events are common and the permafrost active layer thickness is maximum resulting in increased erosion along the river (unpublished data). We hypothesize that the river community is composed of organisms from the surrounding terrestrial environment as well as from upstream freshwater sources, such as glaciers and lakes. Furthermore, due to the relatively short WRT we hypothesize that hydrology rather than local sorting is the dominant factor in shaping the community. We assess to which extent the different communities detected along the river structure the bacterial assemblages in the estuary.

Finally, we test and discuss the potential effects of environmental variables based on multivariate statistical analysis.

## Materials and methods

### Sampling

**Figure 1:**
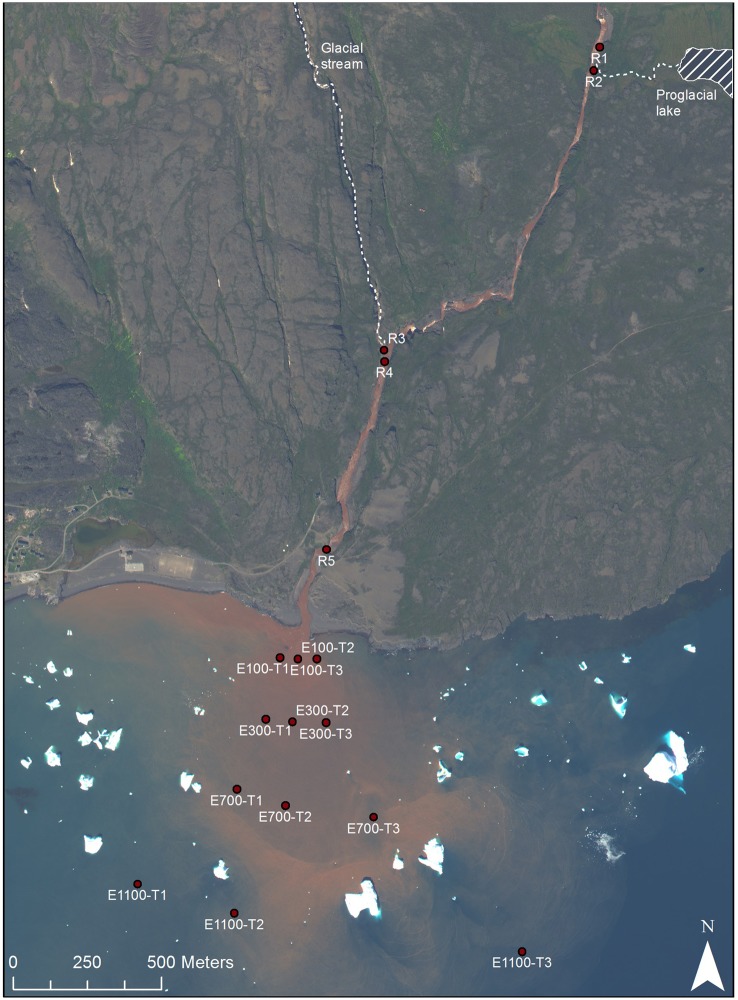
**Sample sites, Disko Island, West Greenland, 69°N (Worldview, 2013)**.

Sampling was carried out in the Red River and its estuary on the Disko Island, West Greenland (69°N) during August 2013 (Figure 1). Around the time of sampling the river flow was 5.7 m^3^s^−1^. The bedrock consists of iron-rich basalt. As the glacier and the stream erode the bedrock the iron precipitates and gives the marked red color of the river. The river drains directly into the Disko Bay and the freshwater and sediments supplied from the river are mixed with the saline bay waters under varying wave influence. A river plume of high concentrations of sediment is often visible indicating how the supplied sediment is dispersed.

Five locations were sampled in the river with three replicates at each site (Figure 1). The top sample (R1) being just upstream of an outlet from an adjacent proglacial lake and the second sample (R2) at the outlet from the proglacial lake. The third sample (R3) being at another outlet to the river supplying water directly from the glacier and the fourth sample (R4) 100 m downstream of R3. Sample R4 was collected at the eastern bank on the opposite site of the upstream outlet from the glacier stream (R3), while all other river samples were collected at the center of the river. The last and fifth sample (R5) was collected close to the river mouth. The distance of each sample to the river mouth is supplied in Table 1. In the bay, sampling was done along three transects perpendicular to the coast (Figure 1). Each transect consisted of four sampling locations at distances of 100, 300, 700, and 1100 m from the river mouth. At each distance two samples were collected, one surface sample at 0.5 or 1 m from the surface and one deep sample 1 m from the bottom. At water depths above 20 m, the deepest water sample was collected at 20 m depth. In transect 1, 100 m into the estuary (E100) the deep sample is missing so that there are only two replicates (transect 2 and 3) of E100 samples. At E700 one sample, which should have been sampled at 20 m, was sampled at 1 m depth, so that there are 4 replicates of surface samples and 2 replicates of deep samples for E700. Water was sampled by grab sampling using sterile 50 ml syringes (Sarstedt, Germany) either collecting water directly from the river or collected from a 5 L Niskin water sampler (KC Denmark, Denmark) that had been filled at the sampled depth. The 50 ml water samples collected in the syringes were forced through Sterivex™ filters (Merck Millipore, MA, USA) and the filters were afterwards partly dried by forcing air from the syringes through the filters. The filters were frozen and kept at −20°C until analysis.

**Table 1:** **Environmental data on Red River and estuary samples**.

**ID**	**Transect**	**Distance m**	**Depth m**	**DOC μM**	**TN μM**	**pH**	**Temperature Degrees C**	**Salinity PSU**	**Oxygen sat Percent**	**Turbidity NTU**	**Particle MD microns**	**Particle TA pixels/vol**	**Particle N number/vol**
R1	R	−2300	0.5	43.6	2.4	7.4	7.4	0.010	103.980	17.660	NA	NA	NA
R2	R	−2300	0.5	344.1	16.9	8.7	12.6	0.023	108.586	1.200	NA	NA	NA
R3	R	−1000	0.5	21.5	3.7	7.3	5.2	0.010	101.105	15.280	NA	NA	NA
R4	R	−900	0.5	65.6	4.8	7.5	6.0	0.010	108.864	15.036	NA	NA	NA
R5	R	−250	0.5	100.9	6.8	7.9	7.5	0.010	106.555	17.273	NA	NA	NA
E100	T1	100	0.5	84.5	6.5	8.0	7.1	23.679	111.213	10.718	55.752	46,016	296.000
E100	T2	100	0.5	166.0	13.6	8.8	6.9	16.205	109.392	19.774	42.329	25,326	237.000
E100	T2	100	4.5	90.3	11.9	8.7	6.8	32.292	113.223	8.209	38.205	16,397	170.000
E100	T3	100	0.5	135.3	13.4	8.9	7.2	26.068	110.465	10.831	67.652	913	4.000
E100	T3	100	2	62.7	4.5	8.7	7.2	26.909	109.804	11.453	46.778	30,034	241.000
E300	T1	300	0.5	79.9	6.7	8.7	7.3	28.489	110.902	10.320	50.871	40,217	296.000
E300	T1	300	10	78.9	4.2	8.4	6.1	32.513	112.439	8.039	35.656	18,483	262.000
E300	T2	300	0.5	60.0	5.3	8.3	6.8	20.794	111.975	14.174	54.712	61,567	338.000
E300	T2	300	8	109.4	8.1	8.6	6.2	32.477	110.957	11.117	40.354	359,091	830.000
E300	T3	300	0.5	192.4	12.1	8.7	7.1	24.422	111.391	13.374	59.102	42,866	261.000
E300	T3	300	4	61.5	4.4	8.4	6.5	32.409	111.552	8.221	35.014	15,617	209.000
E700	T1	700	1	111.9	8.2	8.8	7.2	31.184	110.461	9.113	44.551	15,743	132.000
E700	T1	700	18	76.7	8.8	8.8	2.3	33.219	108.237	8.024	27.317	10,979	138.000
E700	T2	700	1	69.8	5.2	8.5	7.3	31.145	108.917	11.713	76.069	13,631	49.000
E700	T2	700	20	81.7	5.0	8.4	1.8	33.273	108.753	9.478	33.565	9837	132.000
E700	T3	700	0.5	82.1	5.0	8.5	8.5	11.029	112.471	9.169	60.167	90,634	497.000
E700	XX	700	1	46.1	2.5	8.5	7.4	30.898	112.052	13.635	60.931	30,626	142.000
E1100	T1	1100	1	30.2	3.2	8.5	7.1	31.946	110.700	9.694	69.067	15,343	65.000
E1100	T1	1100	20	97.2	8.1	8.8	2.4	33.203	109.182	9.248	29.010	21,687	292.000
E1100	T2	1100	1	115.6	10.4	8.7	7.1	31.733	110.749	11.127	60.402	17,858	82.000
E1100	T2	1100	20	108.1	9.7	8.8	3.0	33.093	111.896	10.633	27.934	16,471	316.000
E1100	T3	1100	1	73.4	6.0	8.7	8.0	26.516	109.069	13.009	58.248	49,641	280.000
E1100	T3	1100	20	171.8	12.1	8.7	2.2	33.245	111.237	8.392	32.262	14,365	208.000

*Distance, Distance to river mouth; Oxygen sat, Oxygen saturation; Particle MD, Particle Mean Diameter; Particle TA, Particle Total Area; Particle N, Particle Number. Particle data was measured in a water volume of 6 mL, vol, measured water volume. Transect XX was sampled outside the three transects*.

Temperature, turbidity, and oxygen saturation were measured at all sites using a YSI 6600-V2 CTD sensor with attached probes (YSI, OH, USA). In the bay, the size (in equivalent spherical diameter, ESD), total area and total number of particles were measured in 6 mL water using a laser sheet camera system, the Pcam (Markussen et al., 2016). Individual water samples were taken at all locations and transferred to new 100 ml polyethylene bottles, frozen as quickly as possible and shipped to Copenhagen for further analysis. The total nitrogen (TN) and dissolved organic carbon (DOC) were determined on a Shimadzu TOC-V total organic carbon analyzer (Shimadzu, Japan) with a TNM-1 total nitrogen measuring unit and pH was measured using a Radiometer Analytical SAC90 autosampler (Hach, CO, USA). DOC measurements were based on triplicate measurements. A standard curve using 1000 ppm sodium hydrogen phthalate with concentrations ranging from 0 to 5 ppm were made and a 100 ppm certified Total Organic Carbon (TOC) standard (SCP Science, QC, Canada) was diluted to 1 ppm for use as reference.

### DNA extraction and sequencing

DNA was extracted from the Sterivex™ filters using the PowerWater© Sterivex™ DNA extraction kit (MO BIO Laboratories, CA, USA), using the protocol provided by the manufacturer. The extracted DNA was stored at −80°C until library preparation.

The nucleic acid concentrations of all samples were assessed by spectrophotometer (Nanodrop® ND-1000, Saveen Werner, SE) to be within the range of 3–5 ng μl^−1^. DNA was then amplified in triplicate using universal prokaryotic primers targeting the variable region V4 of the 16S rRNA gene (Caporaso et al., 2011), forward primer 515F (GTGCCAGCMGCCGCGGTAA) and reverse primer 806R (GGACTACHVGGGTWTCTAAT), using the HiFi polymerase (PCR-Biosystem, UK). The primers were supplied with 12 distinct barcode sequences of 4–6 bases each and combined as differential sets, thus labeling the samples with individual differently tagged sequences. All PCR runs included triplicate positive (*E. coli*) and negative (dd H_2_O) controls. The resulting PCR products (350 bp) were quality controlled by quantification of concentrations using the Qubit® 2.0 dsDNA HS Assay Kit (Life Technologies, CA, USA) and visual inspection of band size following gel electrophoresis. The amplified DNA was then purified using the HighPrep™ PCR size selective carboxyl coted magnetic beads (Magbio, MD, USA). The resulting DNA (average concentration 19.3 ng μl^−1^) was then pooled and ligation of adaptors was performed according to manufacturer's instructions following the Low Sample (LS) Protocol (TruSeq DNA PCR-Free Sample Preparation Guide, Illumina, CA, USA) with minor modifications. Overhangs on the 3′ ends were removed and 5′ ends filled in by end repair, performed as described in the protocol on 1 μg DNA. Size selection was replaced with a clean-up step with magnetic bead based chemistry (HighPrep™ PCR, CleanNA). A volume of 100 μl from the end repair reaction was purified according to manufacturer's instructions and subsequently eluted in 20 μl molecular biology grade water (MO BIO Laboratories, CA, USA). Following this step, the 3′ ends were adenylated (adding an “A” nucleotide) to prevent them from ligating to one another during the ligation reaction. Then adaptors with a “T” overhang were ligated onto the DNA fragments of the two assemblages as described in the protocol. The assemblage was ligated with the AD012 index adaptor (CTTGTA), and then subjected to a clean-up step with purification beads provided in the kit. Quality control of the ligated amplicons (~400 bp) was performed by PCR amplification using primers targeting the index adaptor followed by gel electrophoresis and visual inspection. Finally, the amplicon assemblage was diluted to a concentration of 3.3 ng μl^−1^ and sequenced with MiSeq 250PE (Illumina), adding 30% PhiX DNA. Demultiplexed merged reads are deposited in the NCBI Sequence Read Archive (SRA) database under SRA accession SRP076603.

### Computational analyses

The sequencing data was quality checked using FastQC (Patel and Jain, 2012) and read pairs were merged with the paired-end read merger PEAR (Zhang et al., 2014). Only properly merged reads were used for downstream analysis. Merged reads were processed using Qiime version 1.8.0 (Caporaso et al., 2010a). Demultiplexing with *split_libraries_fastq.py* was performed with quality filtering at phred threshold ≥ 20. Chimeric sequences were removed from demultiplexed data with USEARCH uchime reference-based chimera removal using the Greengenes database from May 2013 as reference (Edgar et al., 2011). Chimera check removed 12.3% of sequences. Operational taxonomic units (OTUs) were subsequently picked based on 97% identity using *de novo* OTU picking, which also includes taxonomy assignment using PyNAST alignment against the Greengenes core set of 16S rRNA sequences (Caporaso et al., 2010b). Sequences only represented once in the dataset were removed, which reduced the dataset with 13.7%.

Shannon indices (Shannon, 1948), Chao1 richness (Chao, 1984) and rarefaction plots were computed using *alpha_rarefaction.py*. Chloroplast sequences were removed and samples were rarefied to the shallowest sample depth of 12,180 sequences per sample with R version 3.1.0 (R Development Core Team, 2008) and R package Vegan (Oksanen et al., 2015). BIOENV analyses were used to assess how well the community structure was explained by environmental variables using non-factorial metadata (Table 1; Clarke and Ainsworth, 1993). For BIOENV analysis the Vegan package was used to create distance matrices of environmental data (Euclidean distances) and community composition (Bray-Curtis distances), which were then compared through Spearman's rank coefficients. DOC, TN, temperature, salinity, oxygen saturation, and turbidity were log transformed prior to analysis. Depth was not included for BIOENV analyses including only river samples as depth was constant and particle data was not included for any analyses including river samples, as the data was not available. LabDSV package in R was used for non-metric multidimensional scaling (NMDS) and indicator species analysis. Indicator species are here denoted indicator OTUs and are defined as OTUs having a higher abundance at one site compared to other sites with indicator values *d* ≥ 0.3 at a significance level of *p* ≤ 0.05. Indicator values are a product of relative abundance of an OTU in samples from one site (between 0 and 1) and the relative average abundance of that OTU across all sites (Dufrene and Legendre, 1997). The used indicator species and indicator OTU concept in this study are not equal to the Indicator Species concept representing species that are markers for certain environmental variables in an ecosystem. NMDS analyses were conducted using Bray-Curtis distance matrices. NMDS stress values are included in **Figure 3**.

## Results

### River system characteristics

Dissolved organic carbon (DOC) and total nitrogen (TN) concentrations in the river were in the same range as those in the estuary (Table 1). River site R2 by the lake outlet had the highest DOC and TN concentrations of all samples in the river and estuary. pH values in the river were slightly lower than in the estuary except for R2, which had a higher pH comparable to the estuary samples. Temperature ranged from 1.7°C at 20 m depth 700 m into the estuary to 12.6°C at river site R2. Temperatures were generally lower in the deep water samples from the estuary compared to the surface samples. Salinity in the river samples was 0.01 PSU for all samples except for R2 where it was 0.023 PSU. The higher salinity in the water from the lake can be explained by the accumulation of ions in the lake due to longer WRT in the lake compared to the river allowing for evaporation of water from the lake. The longer WRT may also explain the higher temperature at site R2. In the estuary, salinity was consistently lower in the shallow water samples compared to deep water samples at the same distance from the river mouth. This was expected from the lower density of the freshwater from the river being mixed into the estuary. Turbidity across all samples, excluding river site R2, ranged from 8.0 to 19.8 NTU, while it was remarkably lower at R2 (1.2 NTU). Camera data from the estuary showed that particle mean diameter was generally higher at shallow depths compared to deep water samples.

### Alpha diversity

**Figure 2:**
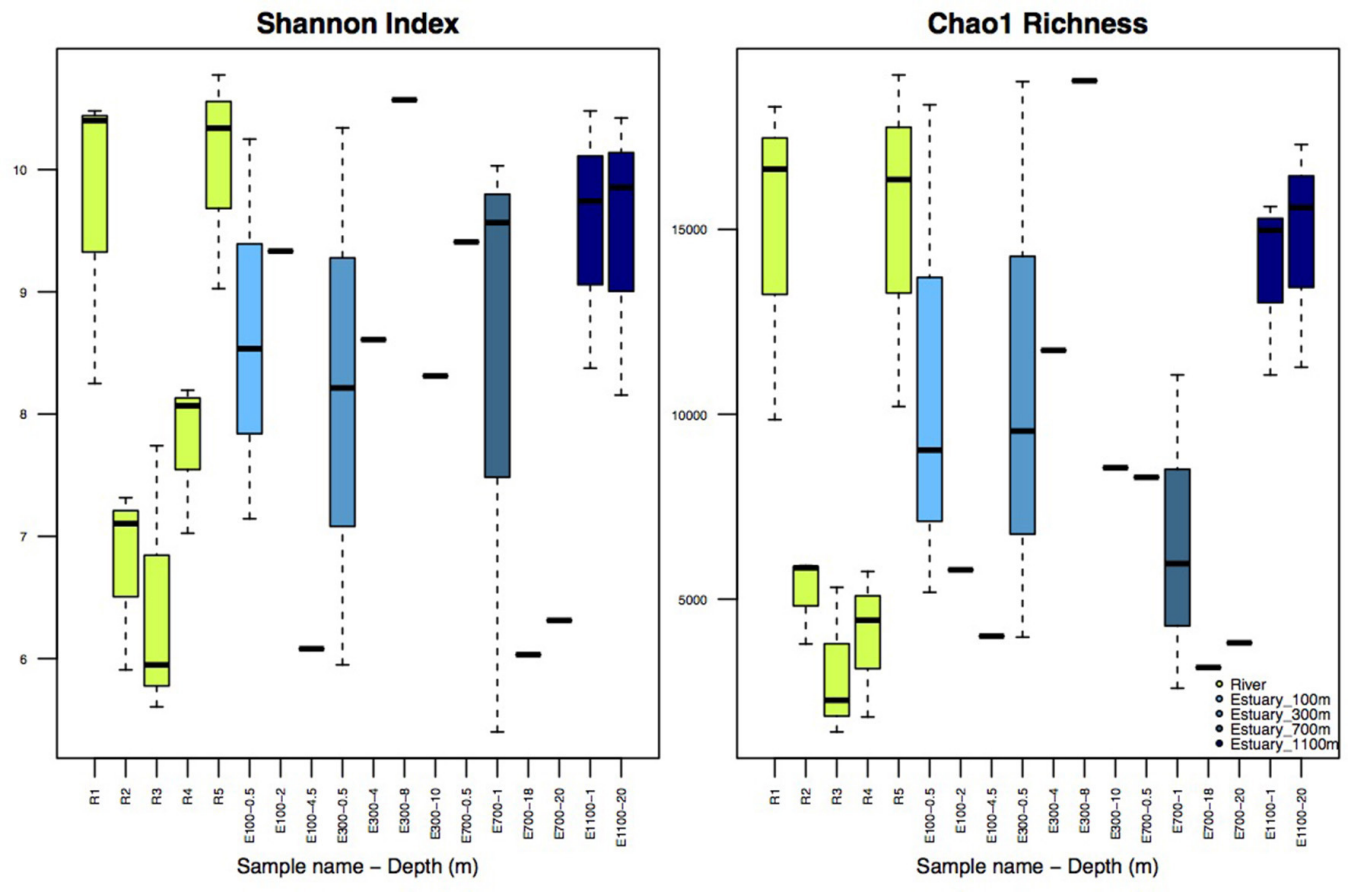
**Shannon Index and Chao1 Richness**. R1–R5 are river samples (*n* = 3), E100–E1100 are estuary samples from 100 to 1100 m into the estuary with varying number of replicates. Replicates are samples from the three different transects and should not be confused with replicates of the same water mass. Depth is indicated after each site name, such that for example E100-0.5 is taken at 0.5 m depth. Sample sites with no replicates (*n* = 1) are indicated without boxes, for sample sites with boxes *n* = 3.

Illumina sequencing of variable region V4 of the 16S SSU rRNA gene from a total of 38 samples resulted in 462,840 individual sequences after rarefaction to 12,180 sequences per sample, which were binned into 63,624 unique OTUs (97% sequence identity). The number of observed OTUs was not exhausted at this level of rarefaction (Supplementary Figure 1). Shannon indices for the river samples ranged from 5.6 to 10.8 and Chao1 richness in the river ranged from 1408 to 19,117 OTUs per sample (Figure 2). The alpha diversity of the bacterial community represented by both Shannon indices and Chao1 richness decreased at R2 and R3, the sites at which the lake and the glacier stream drains into the river (Figures 1, 2). The alpha diversity rose again at R4, ca. 100 m from the glacial input site. At the river mouth (R5), the diversity increased again and reached a similar level to the first river site (R1) upstream of the glacier and lake input sites.

Shannon indices for the estuary samples ranged from 5.4 to 10.6 while Chao1 richness varied from 2589 to 19,021 OTUs (Figure 2). The diversity and richness were higher in the estuary than in the glacier stream and lake input samples, and slightly lower than in the remaining river samples. There was no apparent pattern in the difference in diversity and richness attributed to different depths of the estuary, sample sites or the distance to the river mouth.

### Community composition analysis

**Figure 3:**
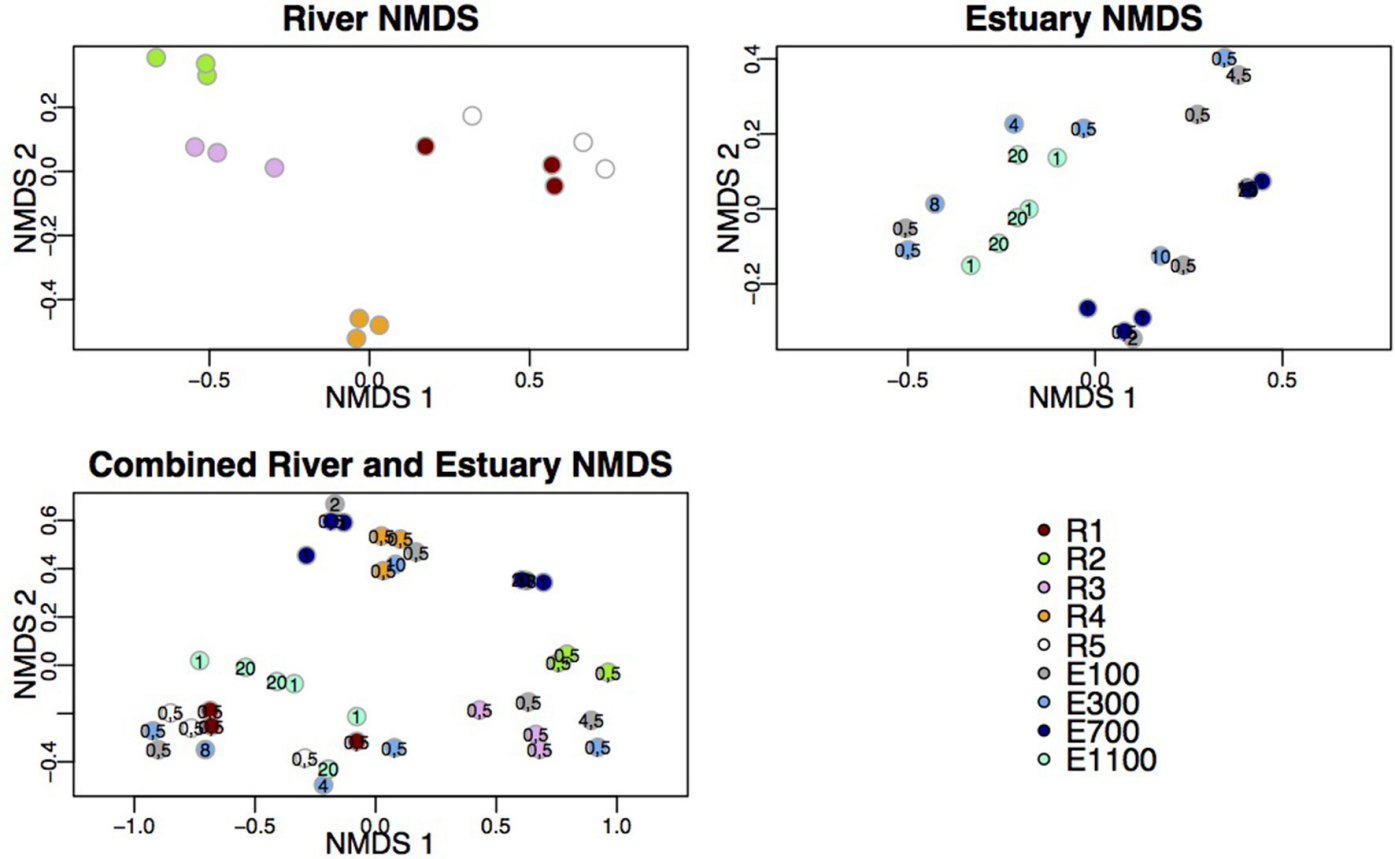
**NMDS plots of river data, estuary data and combined river and estuary data**. Following stress values were obtained: river = 6.49%, estuary = 8.75%, combined = 10.29%. Depths are indicated at the estuary points.

**Figure 4:**
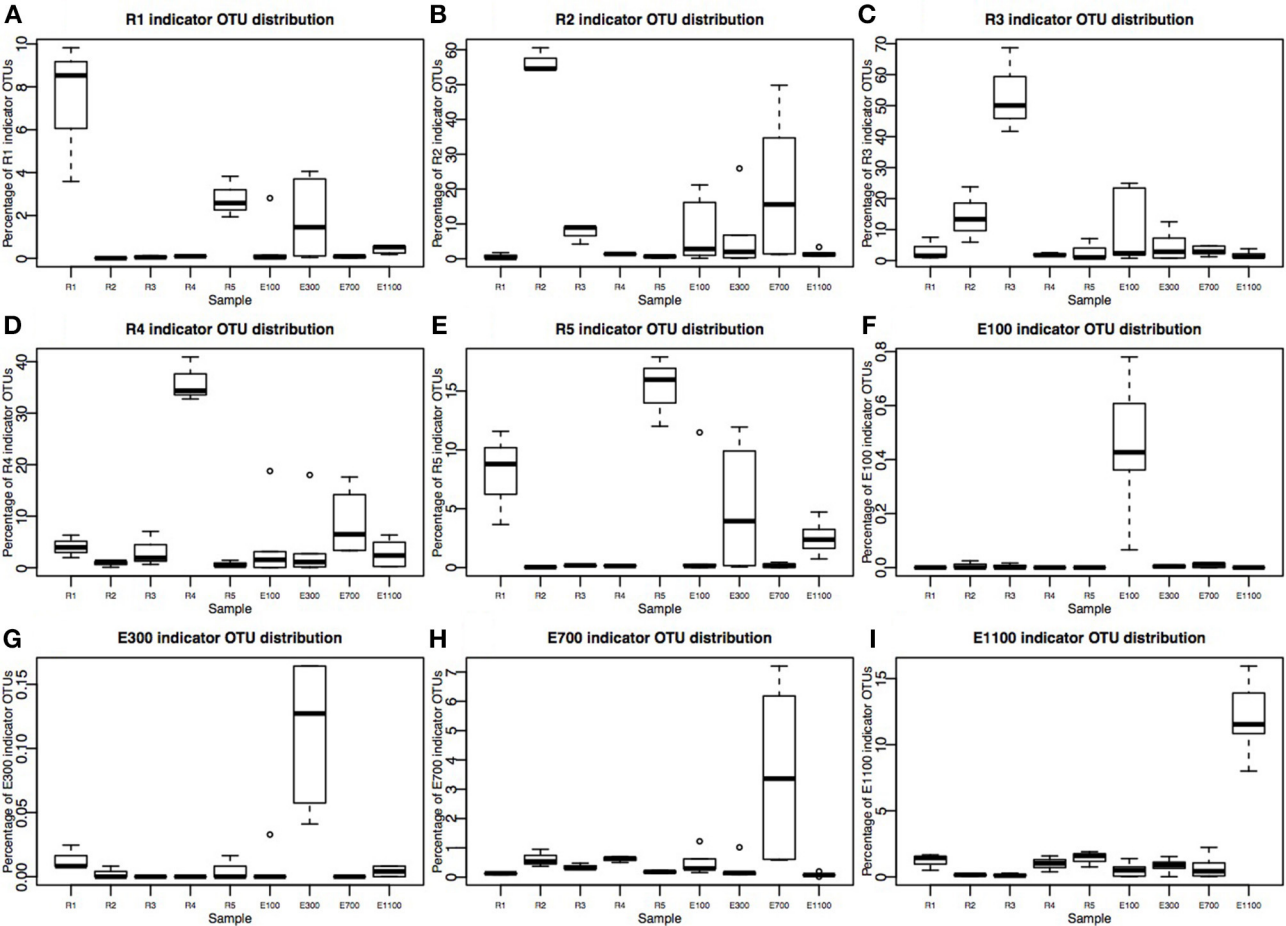
**Percentage of indicator OTU sequences distribution across sample sites**. Please note that Y-axes are different in each plot. **(A–E)** River sites (R1–R5) consist of three replicates corresponding to 36,540 sequences total for each site after rarefaction to 12,180 sequences per sample. **(F–I)** Estuary sample sites E300–E1100 are each sampled in three transects at two depths, making up six samples per distance from the river mouth, corresponding to 73,080 sequences. E100 lacks one sample at transect 1, deep sample, therefore it consists of 60,900 sequences.

The samples from the first site of the river (R1), upstream of the lake and glacier stream outlets to the river, clustered with samples from the bottom site of the river (R5; Figure 3). These two sites also shared a high number of indicator OTUs (Figures 4A,E) and showed similar diversity and richness (Figure 2). The river site by the proglacial lake outlet (R2) clustered with the river site at the glacier stream outlet (R3). These sites, R2 and R3, also had lower diversity than the other river sites (Figure 2).

In the estuary, the bacterial communities clustered according to sample site for the two sites that were farthest into the estuary (E700 and E1100). The samples from the sites closest to the river mouth (E100 and E300) were dispersed across NMDS 1 and 2 (Figure 3). The samples did not cluster according to sample depth. Samples from the sample sites closest to the river mouth (E100 and E300) clustered more closely with river samples than the samples farthest from the river mouth (E700 and E1100).

### Environmental controls

BIOENV analysis showed that the total community as well as the non-indicator and indicator OTUs in the river correlated significantly with turbidity at *p* ≤ 0.05. The strongest correlation was found between the river non-indicator OTUs and turbidity with a Spearman's rank correlation coefficient of 0.586.

BIOENV analysis of the estuary community showed no significant correlations with environmental variables (Table 2).

**Table 2:** **BIOENV analysis of the total, river (*n* = 15) and estuary (*n* = 23) communities and the indicator OTUs and non-indicator OTUs**.

**Community composition subsamples**	**Spearman's rho**	**Environmental variables**
River, Non-indicator OTUs	0.5864	Turbidity
River, Indicator OTUs	0.5473	Turbidity
River, all OTUs	0.5559	Turbidity
Total community, Non-indicator OTUs	0.2021	Temperature, turbidity
Total community, Indicator OTUs	0.1976	DOC, turbidity
Estuary, Indicator OTUs	0.1480	Distance from river outlet, DOC, Salinity, Turbidity
Estuary, all OTUs	0.1015	Distance from river outlet, turbidity, TN, DOC, Total area of particles
Estuary, Non-indicator OTUs	0.0756	Distance from river outlet, turbidity, Total area of particles

### Indicator taxa analysis

**Figure 5:**
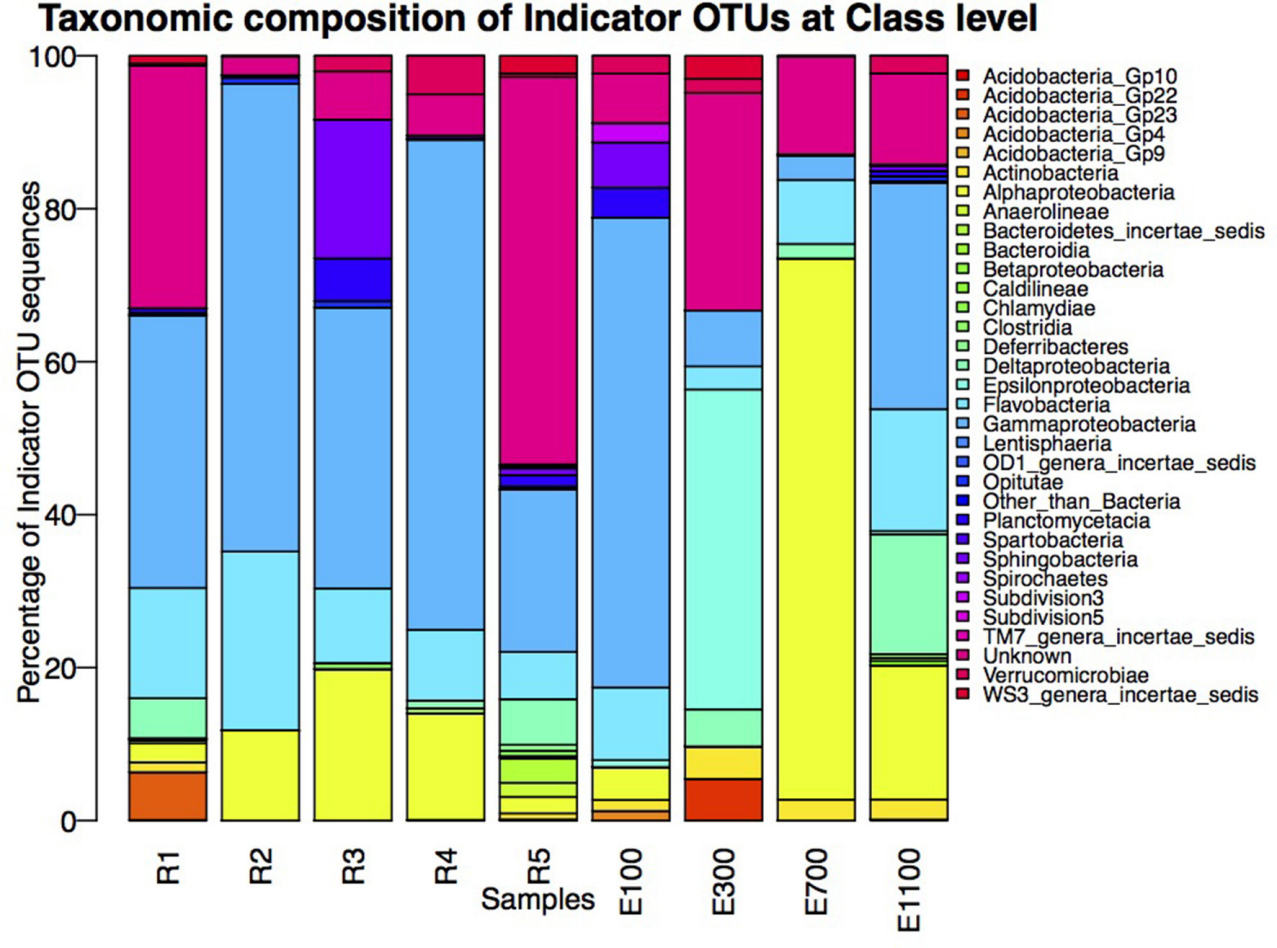
**Taxonomic composition of indicator OTUs at Class level**. Percentages of indicator OTU sequences of each Class are calculated as percentage of the total number of sequences of indicator OTUs from the individual samples.

The number of indicator OTUs in the river ranged from 158 at the second-to-last site of the river (R4) to 678 at the input site from the proglacial lake (R2), which also had the highest percentage of top indicator OTUs (Indicator Value = 1; Table 3). There was a high number of shared indicator OTUs between the top and bottom of the river (Figures 4A,E). Two hundred and eight indicator OTUs from R1 were found at R5 while only 14, 31, and 28 indicator OTUs from R1 were found at R2, R3 and R4 respectively. Taxonomic composition of indicator OTUs at Class level showed similar fractions of Flavobacteria and Gammaproteobacteria across river samples. A greater fraction of Actinobacteria were found in R2-R4 while very few Acidobacteria were found in these samples compared to R1 and R5 where also a higher fraction of Unknown were found (Figure 5). A number of indicator OTU sequences at the uppermost river site (R1) showed similarity to members of *Rhizobiales* isolated from plant roots and soil (Lee et al., 2005) as well as to strict anaerobes such as *Caldilinea, Anaerolineaceae* (Yamada et al., 2006), and *Desulfobacteraceae* (Garrity et al., 2006; Figure 5).

**Table 3:** **Number of Indicator OTUs and Top Indicator OTUs across sample sites**.

**Sample**	Number of Indicator OTUs^*^	Number of Top Indicator OTUs^**^
R1	352	1 (0.28%)
R2	678	34 (5%)
R3	194	5 (2.58%)
R4	158	1 (0.63%)
R5	560	1 (0.18%)
E100	99	0
E300	18	0
E700	43	0
E1100	346	0

* *Including only indicator OTUs with Indicator Value ≥ 0.3 and P-value ≤ 0.05*.

** *Indicator Value = 1*.

Indicator OTUs identified at the outlet from the lake and glacier stream were found in low numbers at the other river sites (Figures 4B,C). The lake outlet site (R2) had the highest number of indicator OTUs and percentage of top indicator OTUs (Table 3). A number of taxa known to be psychrophilic, such as *Moritella* (Urakawa et al., 1998), *Polaribacter* (Gosink et al., 1998), *Oleispira* (Yakimov et al., 2003), *Crocinitomix* (Bowman et al., 2003), and *Psychromonas* (Mountfort et al., 1998) were found among the best matches for the indicator OTUs from the lake outlet, unlike at the other river sites.

The distribution of estuary indicator OTUs showed a different pattern than the river indicator OTUs (Figures 4F–I). The number of indicator OTUs in the estuary was generally lower than at the river sites. An exception to this was the outermost estuary sample (E1100), which had a number of indicator OTUs comparable to the river sites (Table 3). No top indicator OTUs were found in any of the estuary samples, meaning that no OTUs from the estuary were unique to any of the sample sites. The indicator OTUs for each sample site in the estuary were found only in low numbers at the other sites both in the river and the estuary (Figure 4) and the taxonomic composition at Class level was less similar among the estuary samples than among the river samples (Figure 5).

**Figure 6:**
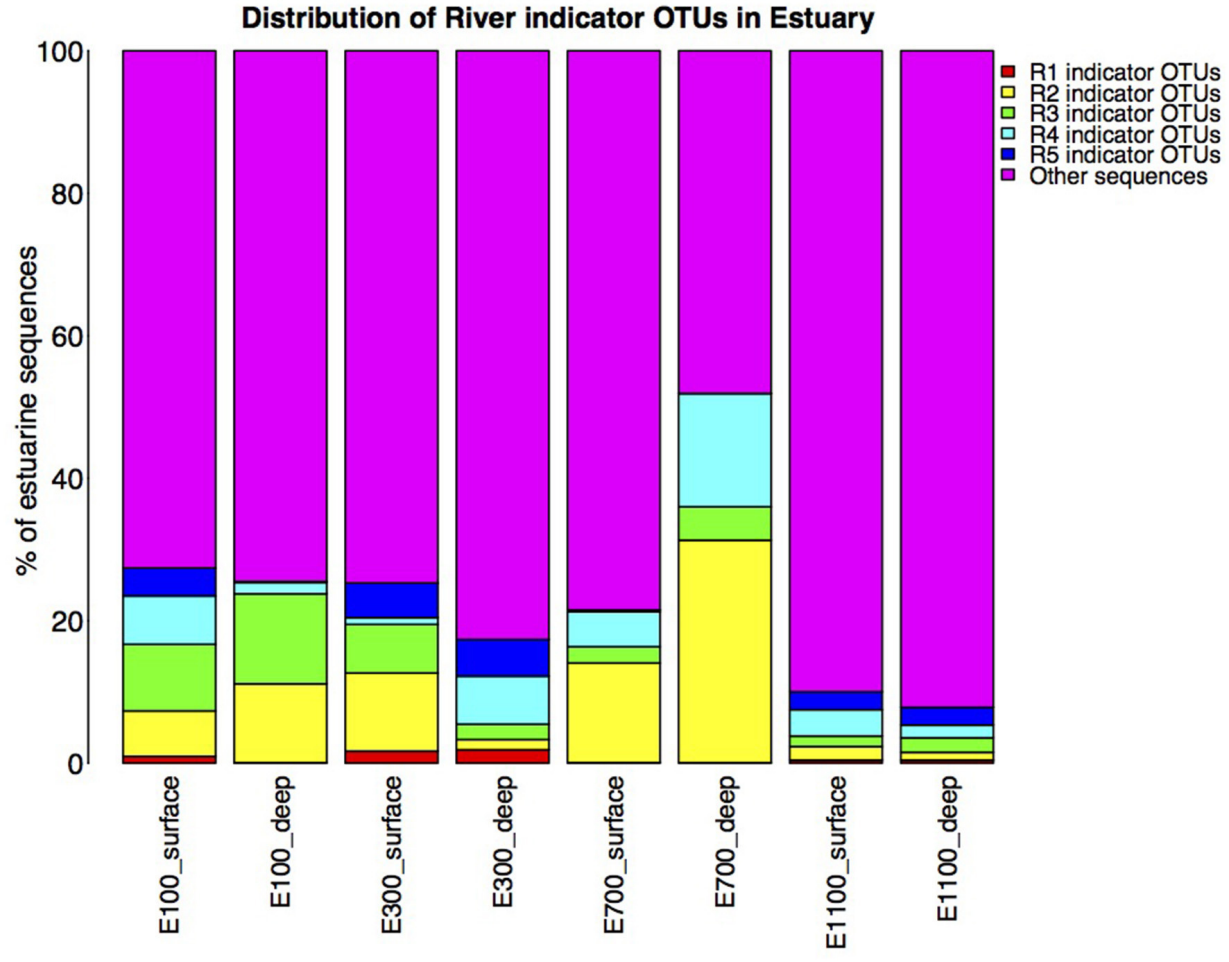
**Distribution of river indicator OTUs in the estuary**. Calculated as percentage of river indicator OTU sequences of total estuary sequences of the estuary site in question. Note that the total number of sequences in each estuary site may differ due to different number of replicates as described in Section Materials and Methods.

On average, the bacterial communities in the estuary were made up of 23% river indicator OTU sequences (Figure 6). There was an overall decreasing contribution of river indicator OTUs in the estuary sites with 26–27% river indicator OTUs closest to the river mouth at E100 sites, 17–25% at E300 sites and 8–10% at E1100 sites. E700 sites were exceptions with 22–52% of the community being river indicator OTUs (Figure 6). Closer to the river mouth at sites E100 and E300 there was a larger fraction of the indicator OTUs from the top of the river (R1) and river mouth (R5), except for the deep sample at E100, where the distribution of river indicator OTUs was similar to the E700 estuary sites (Figure 6). In E700 both in the deep and surface samples R2 (lake outlet) indicator OTUs were more abundant than in the other estuary samples, and were more abundant than indicator OTUs from any other river sites (Figure 6). At the estuary sites farthest from the river mouth (E1100) the samples had the highest fraction of non-river indicator OTU sequences (Figure 6).

## Discussion

### Alpha diversity

Shannon indices for the river samples at the lake outlet (R2) and glacier stream outlet (R3) were comparable to a recent study of 87 small streams and rivers in the La Côte-Nord region of Québec, Canada, where OTUs were clustered with the same method as in the present study (Ruiz-González et al., 2015). The remaining river samples had slightly higher diversity than found in previous studies (Galand et al., 2006, 2008; Crump et al., 2009). The difference from less recent studies is likely due to the difference in the technologies applied and the resulting lower number of sequences in the previous studies. Together with sequencing technologies, which have changed dramatically in the last decade, OTU clustering has shown to have a great impact on the detected alpha diversity (Sinclair et al., 2015). Therefore, the comparison of alpha diversity among studies should be interpreted with care. Our results were obtained with the use of the Qiime pipeline (Caporaso et al., 2010a), which has shown to create a larger number of OTUs when compared to other popular clustering methods (Sinclair et al., 2015). Consequently, we might detect a higher diversity because of the clustering method used.

In an extensive study of freshwater networks, small streams were shown to have higher Shannon indices than larger rivers (Ruiz-González et al., 2015). This was attributed to the common terrestrial origin of the microbial community resulting in an initially high diversity in the small streams originating from the surrounding soil (Ruiz-González et al., 2015). This is in contrast with our results showing less diversity in the glacier stream compared to the main river (Figure 2). The lower diversity might be due to the origin of this stream in a glacier rather than subsurface groundwater and surface runoff as described in the above-mentioned study (Ruiz-González et al., 2015). This notion is supported by the fact that the diversity of the glacier stream outlet (R3) is comparable to those recently described for proglacial lakes (Peter and Sommaruga, 2016). Also, the indicator OTUs from the glacier stream were similar to taxa commonly found in freshwater and marine environments (details not shown). These results emphasize the importance of high spatial resolution for assessing the origin of the metacommunity in a complex freshwater network. In this study, the diversity along the network does not follow a uni-directional pattern (Figure 2). Our results illustrate that the origin and structuring of the microbial community might be very different from one network to another. How glaciers and glacier streams affect the metacommunity of freshwater networks is a highly relevant topic yet to be investigated.

The Chao1 richness in the river samples was higher than in the previous papers focused on large Arctic rivers (Galand et al., 2006, 2008) but comparable to that described in a recent paper using the same sequencing platform (Niño-García et al., 2016).

The drop in alpha diversity at the input sites from the lake and the glacier (R2 and R3, Figure 2) shows that the lake and the glacier stream input less diverse bacterial communities into the main river. Lower diversity in lakes compared with the connected rivers has been attributed to longer WRT in lakes (Crump et al., 2012; Ruiz-González et al., 2015; Niño-García et al., 2016). This is especially pronounced in small streams and rivers, where WRT is too short to allow for local sorting of the bacterial community (Crump et al., 2012; Ruiz-González et al., 2015; Niño-García et al., 2016). Downstream of the input sites, the alpha diversity rises again and by the river mouth reaches a level similar to the first river site (R1) upstream of the input sites (R2 and R3). This shows that the volume of water from the lake and glacier outlets does not dilute the downstream river community. Importantly, it also suggests that the less diverse communities from the lake and the glacier stream are concealed downstream of the input sites by the higher diversity of the main river.

Shannon indices in the estuary samples were higher than those previously described for Arctic estuaries (Galand et al., 2006, 2008). Previous studies of large Arctic rivers show that bacterial diversity and abundance decrease from rivers to estuaries probably due to upstream input from terrestrial sources (Meon and Amon, 2004; Galand et al., 2006, 2008). Our results from a small river support this conclusion by showing a slight decrease in diversity from the main river sites (R1 and R5) to the estuary (Figure 2). The diversity in the estuary sites closer to the river and in the shallow samples could be expected to be higher than more distant and deep estuary samples due to a higher concentration of the river bacterial community, which is not evident from our results (Figures 2, 6). This indicates that although the community structure from the river to the estuary aligns with previous results by showing a directional decrease in diversity, this directional structure cannot be detected further down the network, in the estuary transects. The lack of pattern in diversity and richness attributed to different depths of the estuary samples or the distance to the river mouth could partly be attributed to an insufficient resolution in sample depth. The low resolution might not allow for the detection of a clear plume and different depth zones. While distinct bacterial communities have been found to be associated with the plume and different oceanic zones in an estuary, these results are from sampled oceanic zones several kilometers farther into the ocean than our samples (Fortunato et al., 2012). Estuary samples were previously discussed as harboring a mix of bacterial communities from the river and the coastal ocean with no distinct autochthonous estuary-community (Fortunato et al., 2012), which resembles our results. The high variability in diversity found among the estuary samples suggests a highly heterogeneous community, also expected in such a region where waters of very different chemistry and origin meet. The large variability in diversity and richness seen at the different estuarine sites (Figure 2) could be explained by the sampling of the different water masses, since sequences from the three different transects were pooled together for each estuarine site and depth. The variability of diversity as well as environmental variables seem to lessen farther into the estuary, which could be expected as homogeneity increases as a greater fraction of the estuary is made up of marine waters (Figure 2 and Supplementary Table 1).

### Community composition analysis

Samples from the first site of the river (R1), upstream of the lake and glacier stream outlets, clustered with samples from the bottom site of the river (R5) while the river site by the proglacial lake outlet (R2) clustered with the river site at the glacier stream outlet (R3) as shown by the NMDS plot (Figure 3). R1 and R5 also shared a high number of indicator OTUs (Figures 4A,E) as well as similar diversity and richness (Figure 2). In contrast, R2 and R3 had lower diversity than the other river sites. The NMDS plots, indicator OTU analysis and alpha diversity results imply that waters sourced from the lake and the glacier stream carry different bacterial communities than that of the main river. The larger volume of the main river community then probably masks the lake and glacier stream communities, thus resulting in the close similarity between the sites R1 and R5. The isolation of the R4 samples from other river samples in the NMDS plots may be explained by the difference in sampling at this site, which was closer to the river bank compared to the other river samples. Another explanation might be the imperfect mixing of water from the upstream lake and glacier outlets with that of the main river at this site. The latter seems to be the best explanation since the comparably low number of indicator OTUs found at R4 suggests that this site contains a mixture of the upstream communities rather than a distinct community from the sampling site (Table 3). This agrees with results from a study on an Arctic tundra catchment, showing that streams leaving lakes have decreasing similarity to the lake microbial community as a function of distance (Crump et al., 2007).

The dispersal of estuary samples on the NMDS plots was in accordance with the diversity measures, which were similar within individual sampling sites independent of sample depth (Figures 2, 3). Samples from the sites closest to the river mouth (E100 and E300) clustered more closely with river samples than the samples farthest from the river mouth (E700 and E1100), consistent with a gradual mixing of the river community with a marine community within the estuary environment. Remarkably, R4 river samples clustered more closely with estuary samples than with the other river samples. Indicator OTUs from R4 are present throughout the estuary transects (Figure 6) and the taxonomic composition of samples from R4 has the largest resemblance to that of estuary site E1100 (Figure 5), which might explain the NMDS results (Figure 3). Furthermore, the bacterial community at this site seems to be a mixture of the different river communities as suggested by the indicator OTU results (Table 3). Therefore, the clustering of R4 samples with estuary samples might also reflect the resemblance to the estuary, in which the river communities are also mixed (Figures 4F–I, 6).

Samples from E100 and E300 were more widely dispersed across the NMDS plots than the samples from farther into the estuary, indicating greater heterogeneity of the bacterial communities. This is not unexpected from a region of mixing of largely different water bodies both in terms of physical and chemical variables as well as origin. The NMDS plot did not indicate that the bacterial communities were stratified according to sample depth. The low resolution of samples through the water column might be part of the explanation. However, the results might also indicate a high degree of mixing through the water column of the Red River estuary at the time of sampling. As previously discussed, this may also be explained by the proximity to the river of the estuary samples in this study compared to other studies, where bacterial communities in the estuary were shown to be stratified according to depth (Fortunato et al., 2012).

### Environmental controls

The BIOENV analysis did not show strong correlations between environmental variables and community composition but it did highlight turbidity as a community-shaping factor in the river (Table 2). The bacterial community in a freshwater network fed by glaciers has recently been shown to be structured along the turbidity gradient (Peter and Sommaruga, 2016). The BIOENV results support the idea that the bacterial community in the Red River freshwater network is partly sourced from the glacier. The lower turbidity at R2 (1.2 NTU) compared to an average of 16.3 NTU (*SD* = 1.2) at the other river sites is noteworthy since proglacial lakes are known to have high turbidity (Peter and Sommaruga, 2016). While turbidity of the proglacial lake outlet (R2) is higher than that shown for a non-glacier fed lake in the Austrian Central Alps, it is remarkably low compared to other glacier-fed lakes (Peter and Sommaruga, 2016). This might indicate that the proglacial lake is losing hydrological connectivity to the glacier (Peter and Sommaruga, 2016). It should be taken into consideration that the samples are not taken from the actual lake but several 100m downstream (Figure 1).

The Red River is small in size compared to large rivers previously described such as the Mackenzie River (Galand et al., 2008; Garneau et al., 2009) and the Columbia River (Fortunato et al., 2013). For comparison, the average water flow from August to November in the Columbia River was 2988 m^3^s^−1^ (Fortunato et al., 2013), while the river flow in the Red River around sampling time was 5.7 m^3^s^−1^. An estimated time from top sampling site R1 to the river mouth at R5 is 40 min for the moving water body where the samples are taken. It has been shown that at sites with shorter WRT than 10 days the bacterial community composition was predominantly structured by hydrology (Niño-García et al., 2016). Accordingly, we hypothesized that hydrology would be dominant in shaping the bacterial community in the relatively small Red River with short WRT. Consequently, we did not expect strong correlations between community composition and environmental variables in the river samples. Our study represents a single catchment with short WRT and the results of the BIOENV analysis agrees with previous results by showing weak correlations between the bacterial community and environmental variables (Niño-García et al., 2016).

Salinity has previously been highlighted as a community-shaping factor in estuaries and rivers. For example, the abundance of Alphaproteobacteria, Betaproteobacteria, and Actinobacteria correlated strongly with salinity in the Delaware estuary where a strong negative correlation between Betaproteobacteria and Actinobacteria was shown together with a positive correlation between salinity and Alphaproteobacteria (Kirchman et al., 2005). Salinity, together with temperature, explained 45% of the variation in the community composition in a study of the Mackenzie Shelf (Garneau et al., 2009). Salinity was not identified as a significant factor in the BIOENV analysis. The lack of correlation to salinity in our study is also evident from the NMDS analysis (Figure 3), where R4 river samples cluster with estuary samples despite the large difference in salinity between these environments (Table 1). These results suggest that there may be environmental or hydrological factors other than salinity that explain the observed patterns in taxonomic composition in the study site.

BIOENV analysis of the estuary community showed no significant correlations with environmental variables (Table 2). Also no correlation was found between the bacterial community and spatial variables including distance from the river mouth and depth. The results from the BIOENV analyses indicate that the bacterial community in the estuary is not dispersed according to environmental variables or stratified according to distinct water bodies of riverine or oceanic origin, supporting the results from the diversity assessments (Figure 2) as well as the NMDS plots (Figure 3). Our samples represent a very small fraction of the total estuary; a higher resolution of samples in the estuary might result in more conclusive results.

### Indicator taxa analysis

Indicator OTUs identified at the input sites from the lake and glacier stream were found in low numbers at the other river sites and, therefore, seem to be specific to their respective sources (Figures 4B,C). Notably, of the 678 indicator OTUs from the lake outlet (R2, Table 3), 570 OTUs were not found in the upstream river site (R1) and seem to originate from the proglacial lake. The lake outlet site (R2) had a particularly high number of indicator OTUs and percentage of top indicator OTUs (Table 3). Water bodies with longer WRT have been shown to harbor a less diverse and more differentiated community explained by local sorting of the microbial community (Niño-García et al., 2016). Results from the indicator OTU analysis and diversity of the lake outlet site (R2) show that the lake with a longer WRT has a less diverse and more specialized community compared with the river. A number of taxa known to be psychrophilic, such as *Moritella* (Urakawa et al., 1998), *Polaribacter* (Gosink et al., 1998), *Oleispira* (Yakimov et al., 2003), *Crocinitomix* (Bowman et al., 2003), and *Psychromonas* (Mountfort et al., 1998), were found among the best matches for the indicator OTUs from the lake outlet, unlike at the other river sites.

The short WRT in the Red River network should accordingly result in a low degree of differentiation, which is confirmed by the low number of top indicator OTUs (i.e., OTUs unique for a particular site), which average < 2% in present study (Table 3). Another study of freshwater networks highlighted an average of 11% unique OTUs between different ecosystems as representing a low number (Ruiz-González et al., 2015). These results differ from our study in that they considered many different lakes while our results are obtained from one lake only (Ruiz-González et al., 2015).

Samples from the lake and glacier outlets (R2 and R3) as well as the sample site just after the glacier outlet (R4) had very few Acidobacterial classes compared to the top and bottom site of the river (Figure 5). Acidobacterial classes were shown to be most common in soil compared to the adjacent freshwater network and Acidobacteria in rivers seem to be sourced from the surrounding terrestrial environment (Ruiz-González et al., 2015). The more differentiated lake community thus seems to harbor a lower fraction of organisms from the surrounding soil community compared to the main river. This might partly be explained by the local sorting of the bacterial community in the lake. It could potentially also to be explained by the presence of taxa with different origin than the main river community, as indicated by the large number of indicator OTUs, which are not present in the upstream site. The lower turbidity at the lake outlet site (R2) might also indicate that there is less input of soil to the lake than to the main river, which causes less mass dispersal effect from the surrounding terrestrial environment. The lower turbidity might, however, also be explained by less suspended particles in the lake because of increased sedimentation due to the longer WRT.

A great number of previous studies of riverine microbial communities have suggested and shown that the river communities are influenced by input of microorganisms from surrounding soil environments (Crump and Baross, 2000; Galand et al., 2006, 2008; Crump et al., 2012, 2007; Ruiz-González et al., 2015; Niño-García et al., 2016). Our results support this by showing that potentially soil-related taxa make up a significantly large fraction of the bacterial community making them part of the indicator OTUs of the main riverine bacterial community (Figure 5). Notably, the results also indicate that, along the river, distinct communities may not have the same degree of influence from the terrestrial surroundings. August is a month of high precipitation and increased erosion around the Red River, which would result in a relatively high influence from the surrounding soil community. The influence from soil may be less pronounced in other months as water flow and erosion levels change.

The dominance of soil microbes in freshwater networks has been established in several recent studies, highlighting soil as the origin of the network metacommunities (Ruiz-González et al., 2015; Niño-García et al., 2016). A gradual differentiation of a stream from an upstream lake as a function of distance has been attributed to the origin of the freshwater communities from a terrestrial metacommunity (Crump et al., 2012, 2007). Our results suggest that glaciers may also supply part of the metacommunity resulting in a different structuring pattern of the network. In our case the structuring pattern was not uni-directional throughout the network but rather showed local changes as different bacterial communities were added to the river. This is illustrated in the diversity results (Figure 2) as well as the NMDS plots of the community composition (Figure 3). These results together with the indicator OTU analysis highlight the importance of additional sources of the metacommunity such as glaciers.

The indicator OTUs from the lake-sourced water (R2) can be found in the second highest abundance in the estuary site 700 m into the estuary (E700) (Figures 4B, 6). The taxonomic composition of E700 differs from the other estuary sites and this site contains a high fraction of Alphaproteobacteria (71%) and a relatively small fraction of Gammaproteobacteria (3%) (Figure 5). Of the 678 R2 indicator OTUs 161 are found at the estuary site 700 m into the estuary, where they make up 20% of the sequences at E700 with a higher fraction in the deep samples compared to the surface samples (Figures 4B, 6). This resembles results from the Columbia River, where the estuary samples were comprised of just over 20% riverine community (Fortunato et al., 2012). The distribution of R2 indicator OTUs suggests that although the organisms from the lake do not form a large enough fraction of the community to be notable along the downstream river, they are transported into the estuary where they form a larger fraction of the community. Our results align with the “landscape reservoir” concept proposed for the Toolik lake, Alaska, where rare organisms from the upslope landscape influence downslope bacterial diversity and become dominant in environments with favorable conditions (Crump et al., 2012).

The taxonomy of the nine indicator OTUs from R2 found in high numbers (>100 sequences) in the E700 samples were mostly related to organisms isolated from oceanic environments such as *Marinomonas* (Van Landschoot and De Ley, 1983), *Oleispira* (Yakimov et al., 2003), *Pseudoalteromonas* (Bowman, 2007), *Polaribacter* (Gosink et al., 1998), and *Sulfitobacter* (Sorokin, 1995). Of related non-marine organisms were *Glaciecola*, which was first described as a Gammaproteobacterium isolated from Antarctic sea ice (Shivaji and Reddy, 2014) and *Rhodobacteraceae* known from aquatic environments (Pujalte et al., 2014). The fact that indicator OTUs from the proglacial lake outlet to the river are similar to known marine organisms suggests that these organisms are commonly found in marine environments and that they are not originally known from terrestrial environments. Since it is unlikely that organisms are transported from the estuary to the proglacial lake over 2 km upstream, these organisms in the estuary more likely originate from the upstream freshwater network. Possibly, they become such common organisms in the estuarine and marine environments, that these are the environments from which they have become known. It is well established that bacterial communities found in freshwater networks can be traced from upstream positions in the network (Crump et al., 2012; Ruiz-González et al., 2015; Niño-García et al., 2016). We show that in the Red River estuary the river community can be found in the estuary with an overall decreasing fraction from the river mouth toward the ocean (Figure 6). Interestingly, communities that are not notable throughout the river are transported to the estuary where they seem to become an equally large fraction of the estuary as the main river community (Figures 4B, 6). Distinct communities from the river seem to influence the estuary to different extend, so that communities from certain parts of the river make up notably larger fractions of the estuary at some sites (Figures 4, 6).

“Seed bank” is a term proposed for the fraction of dormant organisms that may be resuscitated when met with different environmental conditions through e.g., dispersal to other ecosystems (Lennon and Jones, 2011). The concept of seed banks was recently extended to freshwater networks where organisms originating in a soil community were proposed as the seed bank for boreal freshwater networks (Ruiz-González et al., 2015). For freshwater networks it was discussed that shallower sequencing depth might lead to the erroneous conclusion that freshwater communities do not derive from a shared pool of terrestrial microbes (Ruiz-González et al., 2015). This could lead to an incomplete understanding of the mechanisms of assembly and the actual linkages and dispersal of microbes between connected ecosystems (Ruiz-González et al., 2015). We show that sampling resolution not only in terms of sequencing depth but also resolution along the network may result in overlooking distinct microbial communities and how these are distributed and linked to the downstream estuary. Our results indicate that not only the terrestrial surroundings but also upstream glaciers may act as seed banks for freshwater networks. While the uni-directional structure in freshwater networks might be a consequence of the numerical dominance of terrestrial OTUs as shown previously (Crump et al., 2012; Ruiz-González et al., 2015) our results suggest that this does not necessarily imply that the bacterial community in a freshwater network has a common origin from microbes from soil. A higher resolution along the river might reveal distinct bacterial communities of different origin and with different composition, which are introduced downstream in the network. These distinct communities, which might be concealed by the numerically dominant terrestrial community along the river, are able to act as seed banks for downstream environments. The different composition of inputs along the river affects the structure of the community, which is not necessarily uni-directional for all freshwater networks as shown in the present study.

Therefore, sampling with the right resolution, both in terms of sequencing depth and the distance between samples along the network, is crucial for understanding the source of microbial communities found in the estuary. This is especially true at times with high precipitation and erosion. Our study shows that with the right resolution, microbial communities can be valuable in understanding transport pathways of meltwater and matter from source to oceans in that they can serve as both tracers as well as indicators of origin in their adaptation to the environment.

The indicator OTUs for each sample site in the estuary were found only in low numbers at the other sites both in the river and the estuary. This pattern is in contrast with the results from the Columbia River, USA, where indicator OTUs from the upper water-column of the estuary (< 56 m depth) showed generalist taxa qualities by having high relative abundance and occurrence in a high number of samples outside their indicator environment when compared to the river environment (Fortunato et al., 2013). Ocean-specific taxa are not expected to be found upstream in the river, which is also apparent from the distribution of estuary indicator OTUs (Figures 4F–I). The low number of indicator OTUs and lack of top-indicators in the estuary show that the different sites in the estuary do not hold distinct communities. These results are in accordance with the results of the BIOENV analysis and NMDS plots, which suggest that the bacterial communities are not dispersed according to environmental or spatial variables, as well as the highly variable diversity measures in the estuary. As previously discussed, samples in the present study are sampled relatively close to the river mouth and a more distinct stratification of the bacterial communities might become visible farther into the estuary.

We expected the estuary sites to contain a mixture of the communities found in the river and the ocean, with more environmental variability closer to the river mouth due to the mixing of river- and sea-water. This is supported by our data, which show greater variance in environmental data closest to the river mouth (Supplementary Table 1) as well as river indicator OTUs from more of the river sites closer to the river mouth at E100 and E300 (Figure 6). Mixing of river and ocean water may result in an allochthonously dominated community shaped by hydrology rather than by environmental selection, also indicated by the NMDS plot (Figure 3) and BIOENV analysis (Table 2). This is supported by the fact that the outermost sample site of the estuary (E1100) had a higher number of indicator OTUs (Table 3) as variability is expected to decrease with increasing distance from the river mouth and the most distant estuary site is expected to contain a higher number of ocean indicator OTUs. This was supported by the taxonomy of the indicator OTUs that were all similar to marine-related taxa at E1100 (details not shown). This site also had a lower fraction of river indicator OTUs compared to the estuary sites closer to the river mouth (Figures 4, 6).

## Conclusions

The bacterial community in the Red River, a small river on the Disko Island, West Greenland, is sourced partly from the surrounding terrestrial environment but also receives distinct microbial communities from a proglacial lake and a glacier stream that harbor lower diversity and different composition than the main river. These input communities are less influenced by terrestrial sources than the main river and the proglacial lake input has a higher fraction of OTUs resembling psychrophilic taxa. The combined community in the river is then mixed with oceanic waters in the estuary, where the indicator OTUs of the river communities made up on average 23% of the estuary community at different sites. While the indicator OTUs from the lake and glacier outlets are not notable in the downstream river they make up large fractions of the community at some sites in the estuary. The bacterial community of the river showed a weak correlation to turbidity while the estuarine bacterial community showed no correlation to environmental or spatial variables. Our results illustrate the added value of examining bacterial communities to better understand and trace the transport of meltwaters from their source to the oceans. Lastly the results show that sampling resolution along the river is crucial for understanding the source of different bacterial communities in a river and estuary system.

## Author contributions

CJ and TM designed the study and sampled, NO performed sample preparations and DNA sequencing, AH performed bioinformatical analyses with contributions from JB and TS and statistical analyses with contributions from MS, AH wrote the manuscript with contributions from MS, TM, NO, BE, and CJ. All authors discussed the results and reviewed the manuscript.

## Funding

This work was supported by the Center for Permafrost (CENPERM) Center no 100 from the Danish National Research Foundation (DNRF100) as well as the Novo Nordisk Foundation Center for Biosustainability.

### Conflict of interest statement

The authors declare that the research was conducted in the absence of any commercial or financial relationships that could be construed as a potential conflict of interest.

## Supplementary material

Supplementary Figure 1 **Rarefaction curve showing number of observed species against sequences per sample**.

Supplementary Table 1 **Statistics of environmental data for estuary samples**.
