# Supplementary figures and images for: Upstream Freshwater and Terrestrial Sources Are Differentially Reflected in the Bacterial Community Structure along a Small Arctic River and Its Estuary
Source: Front Microbiol. 2016 Sep 21;7:1474. doi: 10.3389/fmicb.2016.01474 (PMC5030300; doi:10.3389/fmicb.2016.01474)

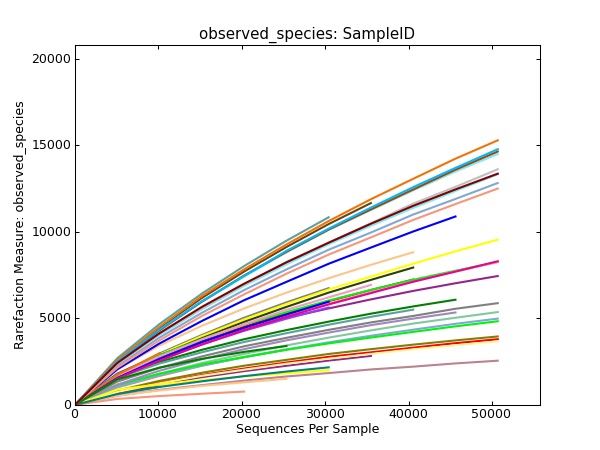

Supplement: Supplementary Figure 1 — Rarefaction curve showing number of observed species against sequences per sample. [file Image1.JPEG]
