# Supplementary material for: Upstream Freshwater and Terrestrial Sources Are Differentially Reflected in the Bacterial Community Structure along a Small Arctic River and Its Estuary
Source: Front Microbiol. 2016 Sep 21;7:1474. doi: 10.3389/fmicb.2016.01474 (PMC5030300; doi:10.3389/fmicb.2016.01474)
Supplement: Supplementary Table 1 — Statistics of environmental data for estuary samples. [file Table1.DOCX]

|  |  | Distance to river mouth | Depth | DOC | TN | pH | Temperature | Salinity | Oxygen Satura-tion | Turbidity | Particle Mean Diameter | Particle Total Area | Particle Amount |
| --- | --- | --- | --- | --- | --- | --- | --- | --- | --- | --- | --- | --- | --- |
| E100 | min | 100 | 0.5 | 62.71 | 4.46 | 8.01 | 6.83 | 16.20 | 109.39 | 8.21 | 38.21 | 913.00 | 4.00 |
|  | max | 100 | 4.5 | 166.02 | 13.63 | 8.93 | 7.22 | 32.29 | 113.22 | 19.77 | 67.65 | 46016.00 | 296.00 |
|  | avg | 100 | 1.6 | 107.76 | 9.97 | 8.62 | 7.05 | 25.03 | 110.82 | 12.20 | 50.14 | 23737.20 | 189.60 |
|  | sd | 0 | 1.75 | 41.91 | 4.20 | 0.35 | 0.19 | 5.85 | 1.51 | 4.41 | 11.76 | 16683.39 | 112.96 |
|  | var | 0 | 3.05 | 1756.11 | 17.63 | 0.13 | 0.03 | 34.26 | 2.28 | 19.48 | 138.23 | 278335386.70 | 12760.30 |
| E300 | min | 300 | 0.5 | 60.05 | 4.23 | 8.34 | 6.11 | 20.79 | 110.90 | 8.04 | 35.01 | 15617.00 | 209.00 |
|  | max | 300 | 10 | 192.35 | 12.08 | 8.73 | 7.29 | 32.51 | 112.44 | 14.17 | 59.10 | 359091.00 | 830.00 |
|  | avg | 300 | 3.92 | 97.03 | 6.80 | 8.53 | 6.65 | 28.52 | 111.54 | 10.87 | 45.95 | 89640.17 | 366.00 |
|  | sd | 0 | 4.21 | 49.98 | 2.97 | 0.18 | 0.48 | 4.96 | 0.59 | 2.55 | 10.30 | 133090.27 | 231.29 |
|  | var | 0 | 17.74 | 2497.51 | 8.81 | 0.03 | 0.23 | 24.64 | 0.35 | 6.51 | 106.17 | 17713019583.00 | 53494.00 |
| E700 | min | 700 | 0.5 | 46.07 | 2.54 | 8.36 | 1.77 | 11.03 | 108.24 | 8.02 | 27.32 | 9837.00 | 49.00 |
|  | max | 700 | 20 | 111.87 | 8.83 | 8.82 | 8.54 | 33.27 | 112.47 | 13.64 | 76.07 | 90634.00 | 497.00 |
|  | avg | 700 | 6.92 | 78.05 | 5.81 | 8.57 | 5.75 | 28.46 | 110.15 | 10.19 | 50.43 | 28575.00 | 181.67 |
|  | sd | 0 | 9.38 | 21.29 | 2.32 | 0.19 | 2.92 | 8.60 | 1.80 | 2.08 | 18.52 | 31318.50 | 158.40 |
|  | var | 0 | 99.55 | 259.61 | 3.54 | 0.04 | 9.84 | 90.77 | 2.97 | 1.83 | 395.85 | 1224798345.00 | 30890.30 |
| E1100 | min | 1100 | 1 | 30.17 | 3.19 | 8.55 | 2.24 | 26.52 | 109.07 | 8.39 | 27.93 | 14365.00 | 65.00 |
|  | max | 1100 | 20 | 171.76 | 12.07 | 8.85 | 8.04 | 33.24 | 111.90 | 13.01 | 69.07 | 49641.00 | 316.00 |
|  | avg | 1100 | 10.5 | 99.37 | 8.22 | 8.72 | 4.97 | 31.62 | 110.47 | 10.35 | 46.15 | 22560.83 | 207.17 |
|  | sd | 0 | 10.41 | 47.00 | 3.22 | 0.11 | 2.71 | 2.59 | 1.13 | 1.63 | 18.40 | 13509.78 | 109.75 |
|  | var | 0 | 108.3 | 2209.35 | 10.36 | 0.01 | 7.33 | 6.70 | 1.27 | 2.65 | 338.64 | 182514105.00 | 12044.97 |
